# How Openness Enriches the Environment: Read More

**DOI:** 10.3389/fpsyg.2019.01123

**Published:** 2019-05-21

**Authors:** Stefanie Trapp, Matthias Ziegler

**Affiliations:** Psychological Diagnostics, Humboldt University of Berlin, Berlin, Germany

**Keywords:** OFCI model, Environmental Enrichment Hypothesis, reading, PIAAC Germany, PIAAC-L

## Abstract

The recently proposed OFCI model and specifically the Environmental Enrichment Hypothesis state that Openness positively influences the development of cognitive abilities (Ziegler et al., 2012). It is assumed that Openness leads to engagement in more learning activities through creating an enriched environment (e.g., reading). However, despite positive evaluations of the OFCI model in general, there is little empirical research on this specific hypothesis. The current paper used a longitudinal design to test the positive impact of Openness on the frequency of reading activities in general and in the specific case of periods of unemployment. PIAAC (Programme for the International Assessment of Adult Competencies) data were used to fit structural equation models. The results show that Openness fosters greater engagement in reading activities over 3 years; a buffering function in case of unemployment could not be found. Theoretical and practical implications are discussed.

## Introduction

The current work builds on the Openness-Fluid-Crystallized-Intelligence model (OFCI model; Ziegler et al., 2012) and the Environmental Enrichment Hypothesis included therein. According to this hypothesis, Openness is assumed to have a positive impact on the development of cognitive abilities. It is hypothesized that higher Openness leads to more learning opportunities by fostering an enriched environment (e.g., by reading). Furthermore, it is assumed that the effect is especially strong in early and late adulthood, because these time periods are characterized by changes (life events like starting one’s first job or retirement) that allow differences in Openness to manifest, facilitating the creation of more learning opportunities. While numerous studies support the notion of developmental relations between Openness and cognitive abilities (Baker and Bichsel, 2006; Von Stumm and Deary, 2012; Ziegler et al., 2012, 2015; Zhang and Ziegler, 2015; Furnham and Cheng, 2016; Wettstein et al., 2017; Ziegler et al., 2018; Trapp et al., 2019; but also see: Von Stumm and Deary, 2013; Hülür et al., 2018), the concrete assumptions of the Environmental Enrichment Hypothesis have rarely been explored. This includes the idea that Openness manifests in activities (e.g., reading) that serve as learning opportunities and in this way enhances cognitive abilities. Moreover, the notion of critical time periods influencing the manifestation of Openness (Ziegler et al., 2012) has also only been tested indirectly. The current study was conducted to fill these gaps.

### Environmental Enrichment Hypothesis

The Environmental Enrichment Hypothesis can be traced back to ideas by Neisser et al. (1996) and Raine et al. (2002). In their study of children’s cognitive development, Raine et al. (2002) showed that higher curiosity as manifested in stimulation-seeking behavior among 3-year-old children goes along with higher cognitive ability at age 11. Earlier, Neisser et al. (1996) had used the term “environmental enriching” to describe the effects of educationally enriched environments on children’s intelligence. Thus, Raine et al. (2002) called the effect found in their study the Environmental Enrichment Hypothesis. They assumed that children create an enriched, stimulating, varied, and challenging environment for themselves by seeking stimulation. This enriched environment is hypothesized to enhance cognitive development. Hence, behaviors like physical exploration of the environment, social engagement with other children, and verbal interaction with adults are thought to enhance young children’s cognitive development.

Ziegler et al. (2012) introduced the OFCI model. They proposed that relationships between Openness and crystallized (Gc) and fluid intelligence (Gf) affect not only immediate performance but also cognitive development. The Environmental Enrichment, Environmental Success and Mediation Hypotheses are probably the most important assumptions in the developmental part of the model: The Environmental Enrichment Hypothesis mirrors Raine et al.’s (2002) proposal described above. The Mediation Hypothesis further assumes that this positive effect of Openness on fluid intelligence also positively affects the development of crystallized intelligence. The Environmental Success Hypothesis assumes that intelligence positively influences the development of Openness.

In their Environmental Enrichment Hypothesis, Ziegler et al. (2012) generalized the effect found by Raine et al. (2002) to adults and the personality trait Openness. Openness is defined as the general willingness to engage with new stimuli, and thus provides a starting point for learning. However, dealing with complex new situations requires Gf. Thus, Openness only leads to learning, and thus the acquisition of Gc, indirectly through mastering complex new information. This mastery is influenced by Gf, which explains why Openness is presumed to be related to both cognitive abilities.

When transferring the Environmental Enrichment Hypothesis into adult life, Ziegler et al. (2012) assumed Openness to be associated with activities that enrich adults’ lives, e.g., “visits to museums, exhibitions, and concerts, or some kind of actual artistic engagement” (p. 180). Trapp et al. (2019) provided first empirical evidence supporting the Environemntal Enrichment Hypothesis in adults. In their study, they considered the activity aspect of the Environmental Enrichment Hypothesis by proposing that mental activities like reading and calculating are important environment-enriching activities. Their results showed that reading and calculating at work and during leisure time do in fact make important contributions to environmental enrichment. These findings suggest that enrichment can take the form of cognitively stimulating content. Furthermore, the study showed that reading professional journals or publications at work and reading diagrams, maps or schematics during one’s leisure time are especially related to Gf. Thus, enrichment does not necessarily have to involve real-life encounters. For adults, it might even be more realistic to assume that mentally enriching one’s environment through reading occurs more often than actually experiencing new situations in real life. Unfortunately, the study by Trapp et al. (2019) did not use longitudinal data, making causal inferences impossible. The current study aims to overcome this deficit and focuses on reading as a means of enriching one’s environment.

### Spelling Out the Environmental Enrichment Hypothesis: Reading Activities

The aim of the current study is to find support for the idea of reading as an important activity behind environmental enrichment. Prior studies (Kraaykamp and Van Eijck, 2005; Trapp et al., 2019) have demonstrated associations between Openness and reading activities. For example, Kraaykamp and Van Eijck (2005) investigated the influence of Big Five personality domains on media preferences and cultural participation in a Dutch sample that included people aged 18–70 (waves 1998–2000 of the Family Survey of the Dutch Population, *N* = 3156). In regression analyses, they found Openness to be a predictor of reading as a preferred leisure activity.

Mussel (2013) could show that Openness strongly overlaps with or even includes traits like typical intellectual engagement (Wilhelm et al., 2003; Arteche et al., 2009), of which reading is an important facet.

As mentioned above, Trapp et al. (2019) showed that reading and calculating at work and during leisure time are important activities behind environmental enrichment. In their study, they used cross-sectional PIAAC data, which include proxies for (a) Openness, (b) Gf, (c) indicators for Gc, and (d) information about the amount of reading and calculating activities conducted at work and during leisure time. The results of structural equation models illustrated that both reading and calculating activities mediated the relation between Openness and Gf and thus also the indirect influence of Openness on Gc (via a mediation by Gf).

In summary, the current study is based on the idea that Openness initiates learning processes that foster Gf and Gc. Thereby, Openness manifests in activities during work or leisure time that enrich a person’s environment. In particular, the current study focuses on reading during leisure time as one example of such activities. Whereas prior research has established that Openness has an influence on a preference for reading (Kraaykamp and Van Eijck, 2005) or a cross-sectional mediation with Gf as an outcome, research focusing on actual reading activities utlizing longitudinal data is lacking. Thus, this developmental interplay between Openness and reading activities is one critical aspect of the current work.

### Environmental Enrichment, Reading, and Unemployment

The previous sections described our general ideas about the role of reading in environmental enrichment. In addition to this general perspective, the interplay of Openness and reading can also be viewed in a more specific context. Specifically, we focus on critical time periods as another hypothesis of the OFCI.

The OFCI model assumes that differences in Openness are more likely to matter in critical time periods. This assumption is based on trait activation theory (Tett and Burnett, 2003; Ziegler et al., 2014), which states that situational variables influence the manifestation of traits and thus their correlations with other variables. Accordingly, the effects of Openness on Gf and Gc should be especially strong in early and late adulthood, periods in which many changes occur (e.g., starting one’s first job or ending a job, starting a family or losing one’s partner). Such life events open up a multitude of options for each person, increasing the opportunity for differences in Openness to manifest. Alternatively, it can also be assumed based on trait activation theory that situational blockers (e.g., a strict work schedule) decrease the likelihood of trait manifestations.

Consequently, the effect of environmental enrichment should be especially strong in periods of life where many changes occur (e.g., starting one’s first job or ending a job, starting a family or losing one’s partner). Losing one’s job is one such major individual life experience (Specht et al., 2011; Boyce et al., 2015) that is associated with negative effects, for example on mental health (Murphy and Athanasou, 1999; Creed and Evans, 2002). Jahoda’s (1982) latent deprivation model explains this negative impact as being due to the loss of latent functions of work, like the imposition of a time structure, regular social contact, and regularly enforced activity.

Creed and Evans (2002) noticed that personality also seems to play an important role in the context of unemployment. They referred to studies showing that some people did not suffer psychologically from being unemployed (Fryer and McKenna, 1987; Hesketh et al., 1987). Creed and Evans (2002) stated that these individuals “have found functional alternatives to accessing the latent functions in order to satisfy their basic psychological needs” (p. 1046). As one example, they named “continuing the pursuit of purposeful activity” (p. 1046). Reading can be seen as such a meaningful leisure activity because it is associated with obtaining new information or new ideas which can help one master one’s new situation after job loss. In addition, literature can be used to learn something new in order to increase one’s chances of getting a new job. Consequently, reading could be a functional alternative to working during periods of unemployment. On the other hand, reading as a solitary activity cannot replace social contact as a latent function of work. However, Waters and Moore (2002) found that activities associated with positive coping responses during unemployment (1) must be meaningful, but (2) can be either solitary or social. In their study, they investigated the role of meaningful leisure activities in a sample of unemployed (*N* = 201) and employed (*N* = 128) Australians. Their findings showed that both solitary and social activities were negatively related to most indicators of deprivation (time structure, shared experience, personal identity, purpose, enforced activity). Consequently, reading can be seen as an important and helpful activity for people who are unemployed. By providing new information and ideas, reading fosters environmental enrichment during unemployment, so that people might not psychologically suffer from deprivation. Thus, reading might help individuals cope with the negative effects of unemployment.

With regard to unemployment *per se*, a study by Viinikainen and Kokko (2012) showed that Openness predicted a higher number of unemployment spells during life, but had no effect on the duration of each individual unemployment spell. Viinikainen and Kokko (2012) stated that “a higher level of Openness might cause individuals to seek out new experience and new challenges, and this would lead to breaks in an individual’s working career” (p. 1214). Thus, more open people might see job change as an opportunity rather than a loss and use the period of unemployment more positively. Findings by Roberts et al. (2003) fit in with this bigger picture by showing that Openness decreases with longer durations of unemployment. This means that Openness is associated with unemployment in two ways. On the one hand, open people seem to be more open to job changes and unemployment. On the other hand, Openness decreases after a longer period of unemployment. The OFCI perspective might provide an explanation for the latter effect, as Openness differences are considered more likely to manifest in critical time periods, including prolonged unemployment. As hypothesized above, reading could be one such manifestation that also acts as a protective factor against the negative effects of unemployment.

In conclusion, some specific hypotheses can be derived from the proposed Environmental Enrichment Hypothesis model. Job loss can be seen as a critical life event. This change can go along with more learning opportunities due to more available time, meaning that Openness could manifest in environmentally enriching activities like reading. Thus, higher Openness should be associated with more reading activities. However, at the same time, job loss in general reduces all activities due to latent deprivation. Hence, the general trend is toward reduced reading activity. In summary, this means job loss should tend to reduce reading activities, but this trend could be buffered by higher Openness.

### Aims of the Study

In accordance with the OFCI model’s Environmental Enrichment Hypothesis, Trapp et al. (2019) showed that reading activities at work and during leisure time mediate the influence of Openness on cognitive abilities. They used the first wave of PIAAC data to test this assumption in a cross-sectional design. The follow-up waves of PIAAC provide an opportunity to investigate the role of reading in the Environmental Enrichment Hypothesis in a longitudinal design. Hence, the current study aims to replicate the findings by Trapp et al. (2019) in a longitudinal design using PIAAC data from 2012, 2014, and 2015 by examining the role of reading activities with regard to environmental enrichment. Because there are only three measurement occasions, the developmental interplay between only two variables can be investigated. Thus, this study will focus only on Openness and its impact on reading during leisure time. Our assumptions with respect to this first research aim are as follows: First, we assume that Openness and reading activities are related cross-sectionally (Hypothesis 1). Second, we assume that Openness will have a longitudinal positive influence on reading activities (Hypothesis 2).

The current study also seeks to examine this effect in the specific context of unemployment. This will test a further hypothesis of the OFCI model. The idea behind this hypothesis is that the relation between Openness and reading activities should be stronger during unemployment due to trait activation. Losing one’s job is generally not seen as a chance to learn something new, but is characterized by symptoms of depression and a general decrease in activities. However, based on the fact that some people do not follow this general trend and do not suffer after a job loss, as well as the idea that meaningful solitary leisure activities (e.g., reading) can be helpful for coping, we hypothesized as follows: first, job loss has a negative effect on reading activities. In other words, reading should decline after job loss, but not among people who remain employed (Hypothesis 3). On the other hand, we propose that this decline in reading after job loss will be buffered among people higher in Openness. Thus, we assume a buffering effect of Openness on this decline in reading activities (Hypothesis 4).

## Materials and Methods

### Sample and Procedure

The current study is based on a secondary analysis of previously published and publicly available data: The German sample of the Programme for the International Assessment of Adult Competencies (PIAAC, Rammstedt, 2013; Zabal et al., 2014, 2016). PIAAC was initiated by the Organization for Economic Cooperation and Development (OECD). In Germany, it was conducted by GESIS – the Leibniz Institute for Social Science. The program started in 2012 with the goal of investigating adults’ competencies. Other factors that might influence the development of such competencies were also examined, such as personality and use of skills like reading as well as information about professional activities. In 2014, a national follow-up study in Germany began. The current study used data from three waves: 2012, 2014, and 2015.

In 2011/2012, PIAAC compared the job-specific competencies of adults in different countries. In Germany, 5465 adults aged 16–65 years took part. The sample was collected using information provided by the municipalities and registry data (Zabal et al., 2014). In 2014, PIAAC anchor persons and their household members were targets of the survey. The sample size here was 7,938 participants, out of which 3,758 subjects had also been tested in 2012 (Zabal et al., 2016). In the following wave in 2015, anchor persons and their partners were of interest. Here, 4,631 people were tested, of whom 3,263 had also been tested in 2012 and 2014 (Zabal et al., 2016). In the current study, only data from PIAAC participants who took part in the survey in 2012, 2014, and 2015 were used. Thus, the final sample size used in this study was *N* = 3263.

In the second part of the current study, only a subsample of these participants were used. We focused on people who had experienced a job loss between 2012 and 2014 and compared them to those who did not experience unemployment in that timespan. Therefore, two groups were built. The first group (*job loss* group) included all people who stated that they were employed full-time (*N* = 120) or part-time (*N* = 71) in 2012 and were currently unemployed in 2014 (*N* = 191). The 85 males and 106 females in this group were between 16 and 65 years of age (*M* = 44, *SD* = 14.96). The second group (*job continuation* group) consisted of all people who stated that they were employed in 2012 as well as in 2014 (*N* = 1831). This group included 981 males and 850 females. Their ages ranged between 19 and 65 years (*M* = 43, *SD* = 10.37).

Data collection for all survey waves was conducted in participants’ homes. Trained interviewers led a personal standardized interview. In the first wave, this also included a background questionnaire (Allen et al., 2013), followed by further computer assessments not examined in the current paper. The current study focuses on data from the personal information questionnaire (e.g., personality and job status). In the last wave, instruments from the National Educational Panel Study (NEPS) were the main focus of the survey. Of these, only the assessment of reading activities was important for the purposes of this study. For more information about the Programme for the International Assessment of Adult Competencies, including information about the data quality standards, see previously published reports about PIAAC (e.g., Rammstedt, 2013; Zabal et al., 2014, 2016) as well as the project’s website^1^.

### Measures

#### Openness Measures

The PIAAC background questionnaire in 2012 included questions about typical habits for dealing with problems and tasks, focusing on the newness of the information. The items were rated on a scale from 1 (*not at all*) to 5 (t*o a very high extent*). The instrument is called *learning strategies* and was used in this study as an indicator of Openness. All six items of the instrument can be found in Table 1.

**Table 1:** Items of 2012 Openness measure (“learning strategies” in PIAAC background questionnaire).

Nr.	Item text
O11	When I hear or read about new ideas, I try to relate them to real life situations to which they might apply.
O12	I like learning new things.
O13	When I come across something new, I try to relate it to what I already know.
O14	I like to get to the bottom of difficult things.
O15	I like to figure out how different ideas fit together.
O16	If I don’t understand something, I look for additional information to make it clearer.

*O11–O16, Openness items in 2012 as named in the models*.

In 2014, a questionnaire developed for the SOEP was used. This questionnaire included a short scale for the Big Five (Lang et al., 2011). Three items assessed Openness [*I see my self as someone who…:* (1) *values artistic/aesthetic experience*, (2) *has a vivid imagination, (3) is innovative, comes up with new ideas*]. Participants had to answer using a rating scale from 1 (*Does not apply at all*) to 7 (*Applies completely*).

It should be noted that we used a scale called *learning strategies* as the Openness measure in 2012. In the second measurement occasion, Openness was measured using the BFI-S (Lang et al., 2011). Unlike the learning strategies scale, which focuses on the intellectual aspect of Openness, the three Openness items in the BFI-S stress the cultural aspect of Openness. This should reduce the autoregressive correlation between the two Openness measures.

#### Measures for Reading Activities During Leisure Time

The background questionnaire for PIAAC Germany 2012 (Allen et al., 2013) included questions about reading during leisure time. The six items concern different sources of information. In the current study, books and newspapers were selected as sources of information because these are the only items measured at both occasions. All questions were answered with regard to frequency (1 = *never*, 2 = *less than once a month*, 3 = *more than once a month and less than once a week*, 4 = *more than once a week, but not daily*, 5 = *daily*).

The 2015 questionnaire included two questions about reading activities during leisure time referring to books/e-books and newspapers (including online newspapers). Both questions were answered on a five-point frequency scale (1 = *daily*, 2 = *at least once a week*, 3 = *at least once a month*, 4 = *less frequently*, 5 = *never*, for analysis the scale was recoded, so 1 *= never* and 5 = *daily*).

#### Information About Job Status

The background questionnaire for PIAAC 2012 (Allen et al., 2013) included a section with job-related questions. One question asked participants to choose from the following list of statements which one best describes their current situation: (1) employed full-time, (2) employed part-time, (3) unemployed, (4) pupil or student, (5) completing an apprenticeship or internship, (6) retired or in early retirement, (7) permanently disabled, (8) in compulsory military or community service, (9) fulfilling domestic tasks or looking after children/family, or (10) other.

In PIAAC 2014, an adapted version of the SOEP personal questionnaire was used. The following two questions were asked regarding job status: “Are you currently employed? Which one of the following applies best to your status? Retirees or individuals in the federal volunteer service (‘Bundesfreiwilligendienst’) who also work in addition to this, please state your job here.” Persons could answer that they were (1) employed full-time, (2) employed part-time, (3) in vocational training, (4) marginally employed, (5) in partial retirement with zero working hours, (6) in voluntary military service, (7) completing a voluntary social/ecological year or federal voluntary service, (8) in a sheltered workshop, or (9) not employed.

### Statistical Analyses

All analyses and data preparation procedures were implemented using R (R Core Team, 2014). In a first step, the data sets for 2012, 2014, and 2015 were merged. Only persons with data in all three measurement occasions were included (*N* = 3263).

Descriptive statistics were calculated using the R package psych (Revelle, 2014). For easier interpretation, the scale concerning reading habits at the second measurement occasion was reverse coded. Construct reliability McDonald’s Ωw was calculated using the R package horst (Horstmann, 2016).

The main analyses were based on testing structural equation models. For these analyses, the R package lavaan 0.5-16 (Rosseel, 2014) was used. Guidelines by Hu and Bentler (1999) for the Comparative Fit Index (CFI ≈ 0.95), the Standardized Root Mean Square Residual (SRMR ≤ 0.09) and the Root Mean Squared Error of Approximation (RMSEA ≤ 0.06) were used to evaluate model fits. In addition, the recommendations of Heene et al. (2011) were applied. Before constructing structural equation models, measurement models for both indicators of Opennness were tested. Measurement invariance between groups was tested according to Chen (2007) recommendations.

Next, correlations between the indicators of Openness and reading activities were estimated. Correlations were considered between reading activities, i.e., reading books/reading newspapers, and Openness measured as (1) a manifest variable using the mean scores of the variables, and (2) a latent variable within a structural equation model. Which reading activities were included into the following structural equation models depended on their relation with the Openness indicator.

**Figure 1:**
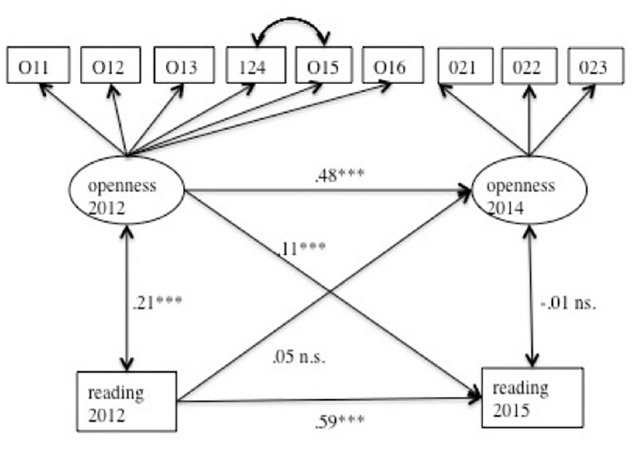
Cross-lagged model for Openness and reading books. Openness and reading books were measured two times each. The model includes autoregressive paths, the correlation path between openness and reading at the same measurement occasion, and cross-lagged paths between Openness and reading at different time points. Openness is a latent variable (loadings on the indicators were left out) and reading a manifest variable. O11–O16, indicators of Openness at the first measurement occasion (see also Table 1), O21–O23, indicators of Openness at the second measurement occasion. ^∗∗∗^*p* < 0.001.

In order to examine the interplay between Openness and reading activities over time, a cross-lagged model was specified (*Model Development*). As can be seen in Figure 1, four important paths were included. First, an autoregressive correlation between the indicators of Openness was specified, representing the development of that trait from 2012 to 2014. The same was done for a manifest reading variable (reading books or reading newspapers). Then, two cross-lagged paths were added to demonstrate the impact of reading on Openness and vice versa. Based on our hypotheses, it was expected that the indicators of Openness and reading would correlate positively within each measurement occasion (Hypothesis 1). This will be tested using the bivariate correlations. Moreover, the Openness indicator at time point 1 was expected to have a positive effect on reading at time point 2 (Hypothesis 2), which was tested with the cross-lagged model just described.

**Figure 2:**
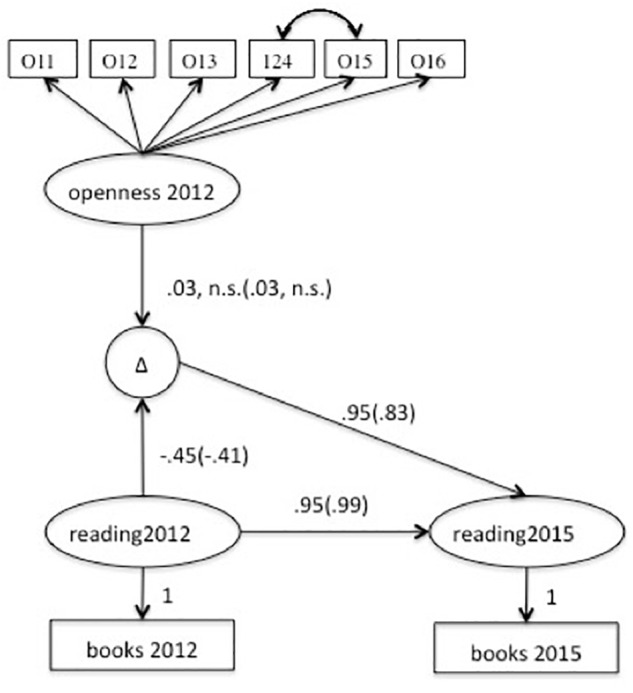
Latent change score model of latent change in reading and the influence of Openness. Δ, latent change in reading; O11–O26, items measuring openness in 2012 (loadings were left out). Non-standardized solution. Model including equal paths for groups.

Afterward, the impact of Openness on reading after job loss was considered. To this end, two groups (*job loss* vs. *job continuation*) were compared using a multigroup latent change score model^2^ (McArdle et al., 2009). Figure 2 displays the Model *Job Loss*. As can be seen, the Openness indicator and both reading measures were modeled as latent variables. The Openness indicator had six items (see Table 1). To create latent variables for the reading measures, the residual variances of the two respective manifest variables were fixed to one minus the commonality of the respective item. These commonalities were derived from a principal component analysis extracting one factor from all reading items. This item communality can be considered a lower bound estimate of the reliable variance. Thus, the difference between one and this communality can be considered an estimate of the unreliable variance (for an example, see Hoppe et al., 2017). This estimate was *1 - h*^2^ = 0.75 for reading books and *1 - h*^2^ = 0.61 for reading newspapers. Next, an autoregressive correlation between the latent variables for the reading measures was added with a fixed regression weight of 1. The residual variance was fixed to zero. Then, the latent change score *delta* for reading was defined using reading 2015 with a fixed loading of 1. Thus, this latent change score includes all reliable differences between the two measurement occassions. To confirm Hypothesis 3, a paired *t*-test was conducted. To estimate baseline effects, reading 2012 was used to predict delta. Importantly, the influence of Openness on this change was estimated by regressing the change score onto the latent variable for the Openness indicator. According to Hypothesis 4, a positive influence of Openness on the change in reading was expected, which would reflect a buffering effect.

In order to test for group differences with regard to the path from Opennss to the change score, a second model was specified and compared. Within this model, the path was restricted to be equal across both groups. Model comparison was based on the difference in CFI, with changes less than ΔCFI ≤ 0.002 indicating no group differences (Meade et al., 2008). These results would falsify Hypothesis 4.

## Results

### Descriptive Statistics and Measurement Models

Descriptive statistics for all variables used in the study to build the measurement and structural models are displayed in Table 2. Tables 4–6 show the intercorrelations of these variables for the subsamples. Here, it can be seen that the sum score of the Openness indicator items was significantly correlated with reading books and newspapers in 2012. In 2015, the correlation with reading newspapers was no longer significant. Thus, Hypothesis 1 was supported with this one exception.

**Table 2:** Descriptive statistics in the general sample, the job loss group, and the job continuation group.

	Whole sample			*Job loss* group			*Job continuation* group		
	*N*	*Mean*	*SD*	*N*	*Mean*	*SD*	*N*	*Mean*	*SD*
*Openness measures*					
O11	3259	3.16	0.82	191	3.06	0.79	1829	3.21	0.79
O12	3263	3.92	0.85	191	3.82	0.90	1831	3.93	0.80
O13	3251	3.59	0.84	190	3.51	0.88	1827	3.60	0.82
O14	3263	3.46	0.92	191	3.51	0.96	1831	3.51	0.87
O15	3254	3.43	0.93	191	3.43	0.98	1824	3.46	0.89
O16	3263	4.05	0.81	191	3.97	0.86	1831	4.09	0.74
O21	3248	4.46	1.90	189	4.35	1.94	1822	4.44	1.87
O22	3259	5.09	1.51	189	5.10	1.55	1830	5.00	1.51
O23	3257	4.92	1.33	191	4.75	1.50	1828	4.94	1.31
OS1	3240	3.60	0.61					
OS2	3243	4.83	1.16					
*Reading measures*					
L1B	3263	3.13	1.44	191	3.18	1.41	1831	3.10	1.44
L2N	3263	4.33	1.06	191	4.22	1.21	1831	4.38	1.04
L2B	3262	3.03	1.45	191	3.19	1.48	1830	3.02	1.45
L2N	3263	4.21	1.17	191	4.09	1.28	1831	4.34	1.10

*O11–O16, Openness items in 2012 (see also Table 1); O21–O23, Openness items in 2014; L1B, reading books in 2012; L2B, reading books in 2015; L1N, reading news in 2012; L2N, reading news in 2015; OS1, sum score for Openness in 2012; OS2, sum score for Openness in 2014*.

The measurement model for the Openness indicator on the first measurement occasion was tested in the general sample as well as in the *job loss* and *job continuation* groups. In all cases, the model was acceptable after adding correlated residuals (see Table 3) between two items that share an analyzing aspect (*to get the bottom of difficult things* and *to figure out how different things fit together*). The model was measurement invariant (configural and metric) for the groups *job loss* and *job continuation* (see Table 3). The six items had correlations between *r* = 0.28 and *r* = 0.59 in the general sample, between *r* = 0.21 and *r* = 0.58 in the *job loss* group, and between *r* = 0.26 and *r* = 0.58 in the *job continuation* group (see also Tables 4–6). Construct reliabilities for these final models were Ω*_w_* = 0.81 in the general sample and Ω*_w_* = 0.81 the *job loss* and *job continuation* groups.

**Table 3:** Model fits.

	Global model fit			Fit indices		
Construct	χ^2^	*df*	*P*	CFI	RMSlEA	SRMR
Measurement models					
Openness Tl in G (1)	409.83	9	< 0.001	0.92	0.12 (90% CI [0.11, 0.13])	0.04
Openness Tl in G (2)	105.58	7	< 0.001	0.98	0.07 (90% CI [0.05, 0.08])	0.02
Openness Tl in JL (1)	20.12	9	0.02	0.96	0.08 (90% CI [0.03, 0.13])	0.03
Openness Tl in JL (2)	17.40	8	0.03	0.97	0.08 (90% CI [0.03, 0.13])	0.04
Openness Tl in JC (1)	255.32	9	< 0.001	0.91	0.12 (90% CI [0.11, 0.13])	0.05
Openness Tl in JC (2)	137.17	8	< 0.001	0.96	0.09 (90% CI [0.08, 0.10])	0.04
Measurement invariance					
Openness Tl conf.	154.505	16	< 0.001	0.954	0.093 (90% CI [0.080, 0.106])	0.037
Openness Tl metric	157.829	21	< 0.001	0.955	0.080 (90% CI [0.069, 0.092])	0.039
Model *job loss* conf.	200.420	38	< 0.001	0.961	0.069 (90% CI [0.056, 0.074])	0.036
Model *job loss* metric	204.194	43	< 0.001	0.962	0.061 (90% CI [0.053, 0.069])	0.037
Single group analysis					
Model *development*	381.18	38	< 0.001	0.96	0.05 (90% CI [0.05, 0.06])	0.04
Model *job loss JL*	33.48	19	0.02	0.96	0.06 (90% CI [0.02, 0.09])	0.06
Model *job loss JC*	164.92	18	< 0.001	0.96	0.07 (90% CI [0.06, 0.08])	0.03
Group comparisons					
Model *job loss A*	207.49	44	< 0.001	0.96	0.06 (90% CI [0.05, 0.07])	0.04
Model *job loss B*	204.20	43	< 0.001	0.96	0.06 (90% CI [0.05, 0.07])	0.04

*df, degrees of freedom; χ^*2*^, chi-square value; p, probability value *of x^*2*^;* SRMR, root mean square residual; RMSEA, root mean error of approximation with 90% confidence interval; CFI, comparative fit index; model (1), model without modifications; model (2), modified model (for modifications, see section “Results”); Tl, at time point 1; T2, at time point 2; in G, general sample; in JL, *in job loss* group; JC, *job continuation* group; conf., model with assumption of configural measurement invariance; metric, model with assumption of metric measurement invariance; model A, with assumption of equality; model B, free estimation of paths.*

**Table 4:** Intercorrelations of variables in general sample.

	O11	O12	O13	O14	O15	O16	O21	O22	O23	L1B	L2B	L1N	L2N	OS1
O12	0.41***													
O13	0.42***	0.41***												
O14	0.31*^∗∗∗^*	0.42***	0.28***											
O15	0.37*^∗∗∗^*	0.43***	0.34***	0.59***										
O16	0.29***	0.45***	0.32***	0.44***	0.46***									
O21	0.15***	0.18***	0.14***	0.12***	0.19***	0.11***								
O22	0.14***	0.2***	0.11***	0.15***	0.22***	0.15***	0.28***							
O23	0.16***	0.24***	0.12***	0.24***	0.27***	0.17***	0.26***	0.40***						
L1B	0.13***	0.16***	0.12***	0.08***	0.12***	0.13***	0.21***	0.08***	0.03					
L2B	0.15***	0.17***	0.16***	0.09***	0.14***	0.14***	0.21***	0.08***	0.01	0.61***				
L1N	0.12***	0.07***	0.06***	0.09***	0.11***	0.12***	0.08***	0.02	0.03	0.19***	0.18***			
L2N	0.07***	< 0.01	-0.01	0.06***	0.07***	0.05***	0.04*	< 0.01	0.03	0.10***	0.12***	0.34***		
OS1	0.60***	0.74***	0.65***	0.73***	0.77***	0.70***	0.21***	0.23***	0.29***	0.17***	0.20***	0.14***	0.06***	
OS2	0.20***	0.28***	0.17***	0.22***	0.31***	0.19***	0.76***	0.74***	0.69***	0.16***	0.15***	0.06***	0.03	0.32***

*O11–O16, Openness items in 2012; O21–O23, Openness items in 2014; L1B, reading books in 2012; L2B, reading books in 2015; L1N, reading news in 2012; L2N, reading news in 2015; OS1, sum score for Openness in 2012; OS2, sum score for Openness in 2014. ^∗^p < 0.05, ^∗∗∗^p < 0.001.*

**Table 5:** Intercorrelations of all variables in the job continuation group.

	O11	O12	O13	O14	O15	O16	O21	O22	O23	L1B	L2B	L1N
O12	0.41***											
O13	0.40***	0.38***										
O14	0.29***	0.42***	0.26***									
O15	0.33***	0.39***	0.32***	0.59***								
O16	0.27***	0.42***	0.28***	0.45***	0.46***							
O21	0.16***	0.17***	0.15***	0.12***	0.19***	0.11***						
O22	0.16***	0.20***	0.10***	0.19***	0.24***	0.16***	0.25***					
O23	0.15***	0.23***	0.11***	0.26***	0.32***	0.19***	0.20***	0.42***				
L1B	0.09***	0.14***	0.12***	0.06***	0.12***	0.09***	0.19***	0.07***	0.01			
L2B	0.14***	0.15***	0.16***	0.06***	0.13***	0.11***	0.21***	0.08***	< 0.01	0.66***		
L1N	0.11***	0.06***	0.02	0.05*	0.08***	0.12***	0.07***	0.01	0.01	0.16***	0.13***	
L2N	0.07***	-0.01	< 0.01	0.01	0.04***	0.04	< 0.01	0.01	0.01	0.09***	0.09***	0.31***

*O11–O16, Openness items in 2012; O21–O23, Openness items in 2014; L1B, reading books in 2012; L2B, reading books in 2015; L1N, reading news in 2012; L2N, reading news in 2015. ^∗^p < 0.05, ^∗∗∗^p < 0.001.*

**Table 6:** Intercorrelations of all variables in the job loss group.

	O11	O12	O13	O14	O15	O16	O21	O22	O23	L1B	L2B	L1N
O12	0.32***											
O13	0.34***	0.35***										
O14	0.31***	0.46***	0.23***									
O15	0.37***	0.40***	0.34***	0.58***								
O16	0.21***	0.34***	0.29***	0.46***	0.43***							
O21	0.02***	0.25***	0.22***	0.17*	0.08	0.18***						
O22	0.16*	0.19***	0.12	0.15*	0.14*	0.06	0.25***					
O23	0.15*	0.29***	0.01	0.29***	0.21***	0.16*	0.32***	0.44***				
L1B	0.13	0.11	0.09	0.08	0.03	0.18***	0.18***	0.05	0.06			
L2B	0.08	0.13	0.16*	0.01	< 0.01	0.06	0.16*	0.07	-0.04	0.53***		
L1N	0.01	0.03	0.17*	0.09	0.07	0.06	0.13	0.01	0.02	0.34***	0.30***	
L2N	0.07	-0.02	0.09	0.02	-0.05	-0.02	0.13	-0.12	0.07	0.16*	0.10	0.30***

*O11–O16, Openness items in 2012; O21–O23, Openness items in 2014; L1B, reading books in 2012; L2B, reading books in 2015; L1N, reading news in 2012; L2N, reading news in 2015. ^∗^p < 0.05, ^∗∗∗^p < 0.001*.

In the second measurement occasion, the Openness items from the BFI-S were used. Because this instrument had only three items measuring Openness, the measurement model can only be specified and model fit cannot be estimated. The item loadings onto the latent variable were λ = 0.42 (*values artistic experience*), λ = 0.66 (*vivid imagination*), and λ = 0.61 (*inventive, full of ideas*). Construct reliability was Ω*_w_* = 0.61 in the general sample (group *job loss:* Ω*_w_* = 0.67, group *job continuation:* Ω*_w_* = 0.63).

Reading newspaper and reading books were correlated with *r* = 0.19 (*job loss* group: *r* = 0.34, *job continuation* group: *r* = 0.16, all *p* < 0.001) in 2012 and with *r* = 0.12 (*job loss* group: *r* = 0.10, *job continuation* group: *r* = 0.09, all *p* < 0.001) in 2015. The low correlation was the reason why no common latent variable was built with these two variables as indicators. Instead, reading books and reading news were analyzed separately.

The autocorrelations across time were positive. The mean scores of the two indicators of Openness were correlated with *r* = 0.32 (*p* < 0.001), and the latent correlation was *r* = 0.49 (*p* < 0.001). Reading books in 2012 and 2015 were correlated with *r* = 0.61 (*p* < 0.001). For reading newspapers, the correlation was *r* = 0.32 (*p* < 0.001). The correlations between constructs and across time were *r* = 0.20 (*p* < 0.001) for the 2012 Openness indicator and reading books in 2015, and *r* = 0.16 (*p* < 0.001) for reading books in 2012 and the Openness indicator in 2014 (see also Table 4). For reading newspapers, the correlation with the Openness indicator at the first time point was *r* = 0.14 (*p* < 0.001, see also Table 4). Both cross-lagged correlations were *r* = 0.06 (*p* < 0.001, see also Table 4). However, reading newspapers and Openness were not correlated at the second measurement occasion. Thus, Hypothesis 1, which assumed that the Openness indicator and reading activities would be related at each time point, was mostly supported. The exception was reading newspapers, which was not related to Openness at the second measurement occasion. Therefore, the following analyses were only conducted with reading books.

### Openness and Reading Activities Across Time

The cross-lagged-model including the Openness indicator and reading activities can be seen in Figure 1. The model fit was acceptable (see Table 3). The autoregressive effect for the Openness indicator was λ = 0.48 (*p* < 0.001). For reading, the autoregressive effect was λ = 0.60. The latent correlation between the Openness indicator and reading at time point 1 was *r* = 0.30 (*p* < 0.001). The residuals for the second occasion were not related (*r* = -0.01, n.s.)^3^. The impact of the 2012 Openness indicator on reading books in 2015 was λ = 0.16 (*p* < 0.001). However, reading in 2012 had no influence on the Openness indicator at the later time point (λ = 0.03, n.s.). Thus, Hypothesis 2 about the positive influence of the Openness indicator on reading at a later time point was supported.

### Openness and Reading Activities After Job Loss

The second aim of the current paper was compare the influence of Openness on reading in two situations, after job loss vs. during continued employment. Hypothesis 3 assumed that reading declines after job loss, and Hypothesis 4 that Openness buffers this decline. The effects are not predicted in the case of continued unemployment.

In the *job loss* group, the frequency of reading books at time point 1 had an average rating of *M* = 3.18 (*SD* = 1.40). At the second time point, the mean was hardly different, *M* = 3.19 (*SD* = 1.48, *t*_190_ = -0.103, *p* = 0.92, Cohen’s *d* = 0.007). In the *job continuation* group, reading rates decreased (*t_1829_* = 2.87, *p* < 0.005, Cohen’s *d* = 0.07) from *M* = 3.10 (*SD* = 1.44) to *M* = 3.02 (*SD* = 1.45). Thus, Hypothesis 3 could not be confirmed. Despite this lack of a mean level change, there could be differential changes, and thus variance in the amount of change, which might be predicted by Openness as stated in Hypothesis 4. This was investigated with a latent change score model (see Figure 2). For both groups, the model fit was acceptable (see Table 3). The change score delta had a significant variance of σ^2^ = 0.96 (*p* < 0.05)^4^ for people who lost their job. For those remained employed, the variance was negative. Thus, we added the constraint to fix the variance of this variable to 1. Thus, despite the lack of mean level change, there were substantial interindividual differences in how reading behavior changed in both groups. While some people read less, others read more. We had hypothesized that the Openness indicator might be related to such differential changes in the case of job loss. To test that hypothesis, we first examined a regression of the latent change score for reading on the Openness indicator in a multiple-group latent change score model in which the path was freely estimated. Model fit was acceptable (see Table 3). However, the path was not significant in either group (unstandardized solution: 0.98, *p* = 0.67, see also Figure 2). Next, this path was restricted to be equal. This model also fit well (see Table 3). The difference in CFI was below the set cut-off. Thus, assuming equal influence did not deteriorate model fit substantially. The path in this restricted model was (*r* = 0.008, n.s.). Thus, Hypothesis 4 could not be supported.

## Discussion

The Environmental Enrichment Hypothesis of the OFCI model (Ziegler et al., 2012) states that the personality trait Openness increases people’s likelihood of encountering new and cognitively stimulating situations, which positively affects the development of cognitive abilities. While the OFCI model in general as well as the Environmental Enrichment Hypothesis had been supported in several studies (Ziegler et al., 2012, 2015; Trapp et al., 2019), there was little examination of possible processes behind this effect. Trapp et al. (2019) found support for the idea that reading activities play an important role in environmental enrichment. In a cross-sectional design, they could show that reading activities at work and during leisure time mediated the impact of Openness on cognitive abilities. Thus, Openness manifests in reading activities, which enriches one’s environment and thereby influences cognitive abilities. The current study sought to replicate the importance of reading activities in a longitudinal design. Therefore, PIAAC data were used to investigate the interplay of Openness and reading activities. In addition, the current study sought to test the idea that Environmental Enrichment is especially likely in times of change. To this end, the impact of Openness on changes in reading activities after job loss was examined. The current findings support the idea that Openness affects reading behavior. However, no buffering effect of Openness on reduced reading activity in times of unemployment was found.

### Relation Between Openness and Reading Activities Across Time

The Openness indicator in 2012 was related to reading books at the same time point as well as at a later time point. For reading newspapers, there were relations between the Openness indicator in 2012 and reading in 2012 and 2015, but not between the Openness indicator in 2014 with reading newspapers in 2015. These findings are not completely in line with the results by Trapp et al. (2019), where the Openness indicator was related to both reading books and reading newspapers. Thus, the effect could be replicated for reading books, but not for reading newspapers. It is hard to explain this failed replication based on the current data. However, given that the Environmental Enrichment Hypothesis assumes that Openness should lead a person to seek out new and most of all stimulating situations, the content of newspapers *per se* might not suffice. This might be different for periodicals or journals. Moreover, the addition of online newspapers to the item might also have had an impact. Such outlets are often consumed using handheld devices over short periods of time. Thus, longer contemplation of the content is not easily possible. The same cannot be said for reading e-books, which requires one to think about the content at least during the time of reading. Thus, future research should pay closer attention to the medium and potentially the situation in which it is used.

The current study adds to the literature by testing the longitudinal effect of Openness on reading activities. Together, this study as well as the study by Trapp et al. (2019) support the idea that reading provides environmental enrichment: Greater Openness fosters willingness to engage in more reading activities at work and during leisure time. According to the OFCI model, more reading activities could serve as learning opportunities, which are assumed to foster the development of fluid intelligence directly and crystallized intelligence indirectly.

### Environmental Enrichment After Job Loss

Besides this general effect of Environmental Enrichment, the OFCI model assumes that this effect changes in magnitude as a result of situational blockers and facilitators. During different life stages, certain life events can occur which act as situational moderators, creating critical time periods. More specifically, Ziegler et al. (2012) stated that the effect should be stronger in early and late adulthood because these life phases are accompanied by numerous changes, allowing differences in Openness to manifest. For example, these life phases are characterized by changes like starting one’s first job/retirement or settling down and starting a family life/losing one’s partner due to death. From the perspective of environmental enrichment, the environment after such a life change is a new one, offering opportunities to arrange one’s environment in a completely new way. More open people are likely to seek out new situations and try completely new ways of handling the situation, while less open people may try to hold on to established ways.

Job loss is one such life event known to be associated with drastic changes [financial constraints and other effects described in Jahoda’s (1981) latent deprivation theory]. It is well known that job loss is associated with negative consequences for one’s life (e.g., higher rate of depression, decreased activities). Like most activities, we expected reading to decrease after job loss as well. Furthermore, we assumed that Openness would be crucial in this context with regard to reading as a meaningful leisure activity and expected the Openness indicator to buffer the decrease in activities like reading after job loss. However, our results revealed neither a decline in reading activity nor a buffering effect of Openness. The latter could be tested due to substantial interindividual differences in changes in reading activity.

Even if Openness does not buffer the decrease in reading activities after job loss, does not follow automatically that Openness cannot buffer negative effects of unemployment *per se*. We supposed that highly open people would see their new situation following a job loss as an opportunity and try out new ways of using their time. They could try out a new hobby or go on a long holiday. This might seem unlikely due to a lack of financial resources. However, there are people who work for only half a year and fill the other half with leisure activities financed by their work during the first half of the year. As these activities are not necessarily associated with more reading, but could also lead to less reading activity in some cases, changes in activities might not be reflected in changes in reading activities. Thus, future studies should include a wider array of Openness indicators.

Another potentially influential variable is the duration of unemployment. In fact, Specht et al. (2011) found a decrease in Openness after job loss depending on the duration of unemployment. They stated that changes in personality could be seen as adaption to one’s new situation after a major life event (e.g., job loss). Perhaps Openness decreases with a longer period of unemployment due to a lack of opportunities to manifest one’s Openness (e.g., having no money to buy interesting books immediately). The current study examined people who were employed at time point 1 and not employed at time point 2, thus spanning a gap of 2 years. Unfortunately, the exact duration of unemployment is unknown. This means that some participants could have been unemployed for nearly 2 years, while others lost their jobs just one or a few months prior. If Openness is only effective for a short time after job loss, the people with longer durations of unemployment would have distorted the results.

### Limitations

The current study has several advantages: First, a large sample with more than 3,000 people was used for the analyses of the general effect of Openness on reading. Second, a longitudinal design was utilized. Nevertheless, there are also several limitations that must be mentioned.

The first limitation is that Openness was measured differently across occasions: the *learning strategies* scale was used at the first measurement occasion and the BFI-S at the second measurement occasion. The two measures differ in focus. The *learning strategies* scale includes questions about habits in learning situations, thus stressing the intellect aspect of Openness, while the BFI-S includes three items emphasizing the cultural aspect (*values artistic experience*; *vivid imagination*; *inventive, full of ideas*). Thus, in the cross-lagged model used in the current study, the change in Openness over time also includes a change in method. This must be taken into consideration when interpreting the rather weak autoregressive path.

The second limitation is that reading books was used as a single indicator for reading behavior and that the exact phrasing of this item changed, as it also included electronic media at the second measurement occasion. Thus, as was the case for Openness, content validity might have changed. This along with the narrow scope of the reading items used reduces the measure’s generalizability to reading in general. Thus, the results should be interpreted with this limitation in mind.

## Conclusion

The current study can be seen as a further extension of the study by Trapp et al. (2019). In that paper, it was found that reading acted as a mediator between Openness and fluid intelligence. While this generally expanded our understanding of environmental enrichment, the cross-sectional nature of the data limited its generalizability. To ensure that reading is influenced by Openness, the current study considered two measurement points. Thus, the development of Openness and reading over time as well as cross-lagged influences were examined. The results supported the impact of Openness on the development of reading activities.

Moreover, the influence of life events was considered as a further component of the OFCI model. We expected that reading activity would decrease after job loss, whereas Openness would buffer this negative trend. We found that reading activities changing on the individual level. The buffering effect of Openness was not supported. As discussed before, we would not conclude that Openness has no buffering effect on reduced activities in general. We assume that (A) Openness manifests in activities other than reading after job loss, (B) that the effect is only relevant for a certain time period after job loss (e.g., only in the first months), and (C) that individuals’ financial situation might prevent differences in Openness from manifesting.

## Ethics Statement

This is a secondary analysis of previously published and publicly available data.

## Author Contributions

ST and MZ analyzed and wrote the manuscript.

## Conflict of Interest Statement

The authors declare that the research was conducted in the absence of any commercial or financial relationships that could be construed as a potential conflict of interest.

## Footnotes

1 http://www.gesis.org/en/en/piaac

2 Thanks to the reviewer for this suggestion.

3 Thanks to the reviewer for suggesting a model with *arts* and *ideas* as a single indicator for Openness at the second occasion. The results can be found in the Supplementary Material. Implications for our hypothesis stay the same regardless of the indicator of Openness used.

4 Standardized solution.
