# Supplementary material for: How Openness Enriches the Environment: Read More
Source: Front Psychol. 2019 May 21;10:1123. doi: 10.3389/fpsyg.2019.01123 (PMC6536890; doi:10.3389/fpsyg.2019.01123)
Supplement: Supplementary file 1 [file Table_1.docx]

Supplemental material

**
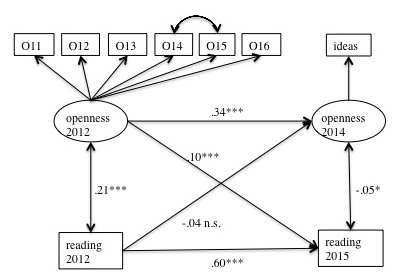
**

*Figure S1*: Cross-lagged model for Openness and reading books with *ideas* as single indicator for Openness 2014. Openness and reading books were measured two-times each. The model includes autoregressive paths, correlation path between Openness and reading at the same occasion, and cross-lagged paths between the Openness and reading at different time points. Openness is a latent variable (loading on the indicators were left out) and reading a manifest variable. Note that, the model is equal to *Model Development* (see Figure 1), but the item *ideas* (BFI-S, see also section *Openness* *measures*) was used as single indicator for Openness at 2014. The model fit was acceptable: *N* = 3263, *χ^2^* =280.277, *df* = 23, *p* <.001, CFI = .97 , RMSEA = .06 ( 90%CI [ .05 , .06] ), SRMR = .03.

*Note.* O11 – O16 = indicators of Openness at the first occasion (see Table 1).

** p < .05, *** p < .001*

*
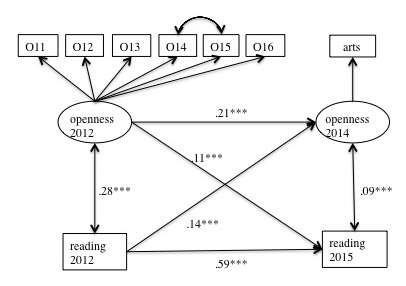
*

*Figure S2*: Cross-lagged model for Openness and reading books with *arts* as single indicator for Openness 2014. Openness and reading books were measured two-times each. The model includes autoregressive paths, correlation path between Openness and reading at the same occasion, and cross-lagged paths between the Openness and reading at different time points. Openness is a latent variable (loading on the indicators were left out) and reading a manifest variable. Note that, the model is equal to *Model Development* (see Figure 1), but the item *arts* (BFI-S, see also section *Openness* *measures*) was used as single indicator for Openness at 2014. The model fit was acceptable: *N* = 3263, *χ^2^* =242.261, *df* = 23, *p* <.001, CFI = .97 , RMSEA = .05 ( 90%CI [ .05 , .06] ), SRMR = .03.

*Note.* O11 – O16 = indicators of Openness at the first occasion (see Table 1).

**** p < .001*
